# Analysis of Factors Associated with Hiccups Using the FAERS Database

**DOI:** 10.3390/ph15010027

**Published:** 2021-12-24

**Authors:** Ryuichiro Hosoya, Reiko Ishii-Nozawa, Kota Kurosaki, Yoshihiro Uesawa

**Affiliations:** 1 Department of Medical Molecular Informatics, Meiji Pharmaceutical University, 2-522-1 Noshio, Kiyose, Tokyo 204-8588, Japan; d196955@std.my-pharm.ac.jp; 2 Department of Pharmacy, Japanese Red Cross Musashino Hospital, 1-26-1 Kyonan-cho, Musashino, Tokyo 180-8610, Japan; 3 Department of Clinical Neuropharmacology, Education and Research Unit for Comprehensive Clinical Pharmacy, Meiji Pharmaceutical University, 2-522-1 Noshio, Kiyose, Tokyo 204-8588, Japan; reiko-in@my-pharm.ac.jp

**Keywords:** hiccups, adverse effects, sex characteristics, pharmaceutical database, nicotine

## Abstract

In this study, we used the large number of cases in the FDA adverse-event reporting system (FAERS) database to investigate risk factors for drug-induced hiccups and to explore the relationship between hiccups and gender. From 11,810,863 adverse drug reactions reported between the first quarter of 2004 and the first quarter of 2020, we extracted only those in which side effects occurred between the beginning and end of drug administration. Our sample included 1454 adverse reactions for hiccups, with 1159 involving males and 257 involving females (the gender in 38 reports was unknown). We performed univariate analyses of the presence or absence of hiccups for each drug and performed multivariate analysis by adding patient information. The multivariate analysis showed nicotine products to be key suspect drugs for both men and women. For males, the risk factors for hiccups included older age, lower body weight, nicotine, and 14 other drugs. For females, only nicotine and three other drugs were extracted as independent risk factors. Using FAERS, we were thus able to extract new suspect drugs for drug-induced hiccups. Furthermore, this is the first report of a gender-specific analysis of risk factors for hiccups that provides novel insights into drug-induced hiccups, and it suggests that the mechanism responsible is strongly related to gender. Thus, this study can contribute to elucidating the mechanism underlying this phenomenon.

## 1. Introduction

Hiccups are occasionally experienced by most individuals, and they are mainly caused by diaphragmatic myoclonus [1]. Although hiccups are rarely life-threatening, they often reduce the quality of life of individuals who experience them, which impacts the patient’s normal life. Intractable and persistent hiccups can disturb verbal communication, sleep, eating, and drinking, and in severe cases can result in weight loss, exhaustion, anxiety, and depression [2]. Control of these symptoms is particularly important clinically speaking because treatments may be disturbed when hiccups occur as an adverse effect.

Hiccups are a brief involuntary twitching of the diaphragm muscles accompanied by the coordinated contraction of the glottic-closure group of muscles [1]. It has been reported in animal experiments that the glossopharyngeal nerve (the 9th cranial nerve), the vagus nerve (the 10th cranial nerve), the nuclei of the solitary tract, the nucleus ambiguus, and the phrenic nerve are all involved in the afferent and efferent pathways of the hiccup reflex arc [3]. Although the exact mechanisms of the central link of the hiccup reflex arc are not clear, in their review, Nausheen et al. reported that the hiccup reflex arc is potentially mediated by central neurotransmitters (γ-aminobutyric acid (GABA), dopamine, and serotonin) and peripheral neurotransmitters (epinephrine, norepinephrine, acetylcholine, and histamine) [4,5].

Based on their duration, hiccup bouts are classified as transient (lasting for less than 48 h), persistent (lasting less than 1 month but more than 48 h), or intractable (lasting more than 1 month) [2]. No reports of gender differences or physical information about the common hiccups experienced by normal people are available. However, the incidence of intractable and persistent hiccups is predominant in male patients. Although Lee et al. reported a male predominance in peripheral hiccups, no gender differences were reported in hiccups occurring as a result of central nervous system disorders in their meta-analysis [6].

Several studies have investigated drug-induced hiccups. In particular, cisplatin and dexamethasone—used in chemotherapy for cancer treatment—have been reported as important suspect drugs [7,8,9,10]. As there have been no comprehensive risk factor reports on the association between hiccups and medications, we analyzed the medications and patient information associated with hiccups using the Japanese Adverse Drug Event Report database, JADER, through a data mining approach. As a result, we succeeded in extracting key suspect drugs, including dexamethasone and several anticancer drugs, as risk factors for hiccups. In addition, we found a novel risk factor—tall stature—from patient information associated with hiccups [11]. In an exploratory study of risk factors for hiccups in hospitalized patients, we found an association between hiccups and low body mass index (BMI), symptoms of nausea, vomiting, and chemotherapy-related drugs [12,13]. However, the number of reports of hiccups in JADER is relatively small—about 150 cases—and it is limited to reports from within Japan. Furthermore, the hospitalized-patient data came from a single-center retrospective study, and they may have contained various biases. To extract risk factors that are more closely related to hiccups, we therefore concluded that it was necessary to employ a larger, global database. Consequently, we decided to investigate drug-induced hiccups using a U.S. Food and Drug Administration (FDA) adverse-event reporting system (FAERS) database that collects adverse-event reports from around the world. FAERS contains more than 1 million reports and is the largest database of adverse-event reports in the world [14]. Using FAERS, we thus expected to be able to extract important risk factors and suspect drugs that were not obtained from JADER. The purpose of this study was therefore to extract risk factors for hiccup reversal and patient information using FAERS. In addition, although hiccups are more common in males, there have been no reports on the risk factors for each gender separately. As a secondary outcome of this study, we also decided to extract risk factors for hiccups by sub-analyses of the two groups of men and women separately.

## 2. Results

### 2.1. Database

**Figure 1:**
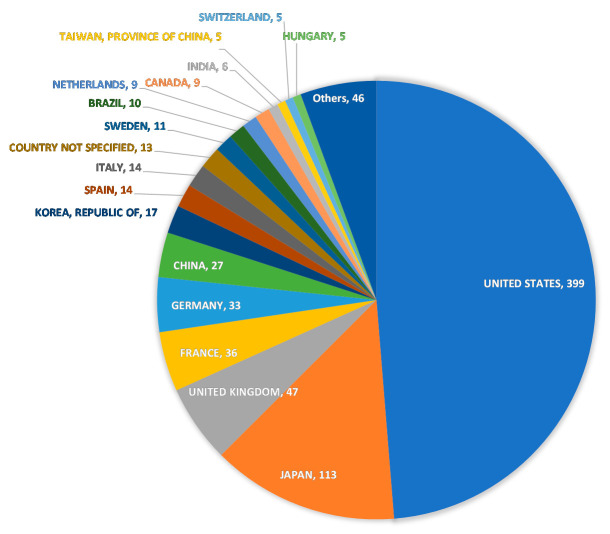
Number of hiccup cases reported by country in the data table for hiccup cases only. The country name and the number of reports (cases) are indicated.

The total number of adverse drug reactions reported between the first quarter of 2004 and the first quarter of 2020 was 11,810,863. Of these, we used 8,184,203 (1,410,565 cases), for which there was a clear relationship between drug use and adverse drug reactions, as the analysis table. In the analysis table, hiccups occurred in 1454 reports (843 cases). The reported rate of hiccups was approximately 0.06%. The top three countries by number of reports for hiccups were the United States, Japan, and the United Kingdom, with 399, 113, and 47 reports, respectively (Figure 1).

### 2.2. Patient Information

**Figure 2:**
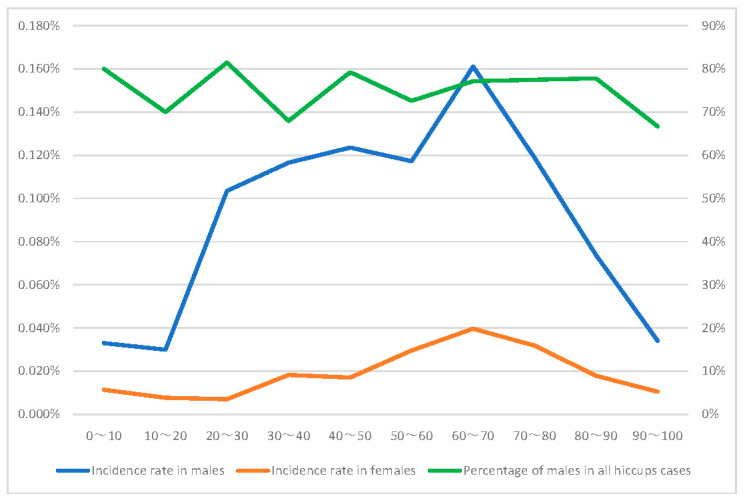
Male and female incidence rates and the percentage of males in cases of hiccups. The graph shows the age on the horizontal axis and the incidence rate (%) on the left vertical axis. The percentage of males in all hiccup cases is shown on the right vertical axis.

The relationships between patient information and hiccups in the data tables for all reports, for males only, and for females only are shown in Table 1, respectively. Each item included missing values; however, the analyses were performed using data without missing values. The sum of the male and female tables stratified by gender did not match the sum of all the tables. In the group with hiccups, 843 cases were divided into two groups according to gender, with 622 cases being male and 193 cases being female. The gender in 28 cases was unknown. In the analysis table, 74% of the reports of hiccups involved males, significantly more than the number that involved females (*p* < 0.001). The hiccup incidence rates in the separate male and female data tables and the percentage of all hiccups reports that are male are plotted as functions of age in Figure 2. Incidence was the greatest between the ages of 60 and 70 years in both males and females, and it was higher for males than for females of all ages. The percentage of males in all hiccup cases was approximately independent of age. The mean ages of the hiccup and non-hiccup groups were 59.28 ± 0.76 and 54.63 ± 0.04 years, respectively. The hiccup group was significantly older (*p* < 0.001). The mean weights of the individuals in the hiccup and non-hiccup groups were 74.37 ± 1.36 kg and 73.11 ± 0.01 kg, respectively, i.e., there was no significant difference (*p* = 0.36).

In the male data table, hiccups were reported in 622 cases (Table 1). The mean ages of the hiccup and non-hiccup groups in the male table were 59.35 ± 0.85 and 57.59 ± 0.03 years, respectively, i.e., the hiccup group was significantly older (*p* = 0.04). Similarly, the mean weights of the individuals in the hiccup and non-hiccup groups were 75.81 ± 1.58 kg and 78.51 ± 0.06 kg, respectively, with no significant difference (*p* = 0.09).

In the female data table, hiccups were reported in 193 cases (Table 1). The mean ages of the hiccup and non-hiccup groups in the female table were 59.02 ± 1.59 and 52.61 ± 0.02 years, respectively; again, the hiccup group was significantly older (*p* < 0.001). Similarly, the mean weights of the individuals in the hiccup and non-hiccup groups were 69.72 ± 2.88 kg and 69.08 ± 0.05 kg, respectively, with no significant difference (*p* = 0.823).

### 2.3. Hiccup-Inducing Medications

**Figure 3:**
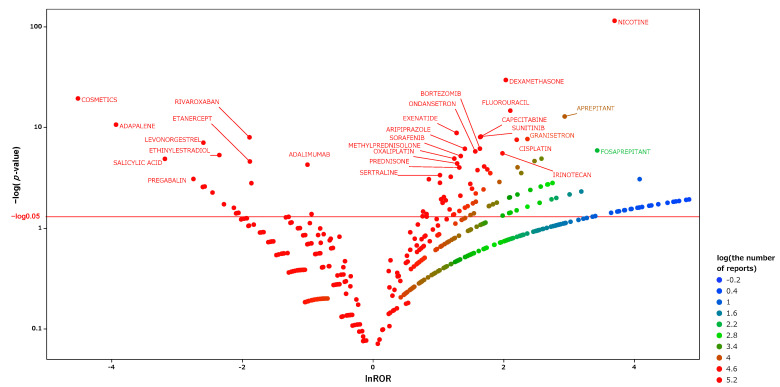
Medicines associated with hiccups. This figure shows the relationship between adverse effects such as hiccups and the medicines suspected to have caused them. We constructed this volcano plot by plotting the negative logarithm of the *p*-value (−Log_10_*p*) from Fisher’s exact test on the *y*-axis and the natural logarithm of the ROR (lnROR) on the *x*-axis. The red line shows the baseline *p* = 0.05. The colors of the individual points represent differences in the log of the number of reports for each drug. In this scatter plot, the signal is larger for the points (drugs) plotted in the upper right corner. The blue-to-red colors represent the number of times an adverse effect was reported.

**Figure 4:**
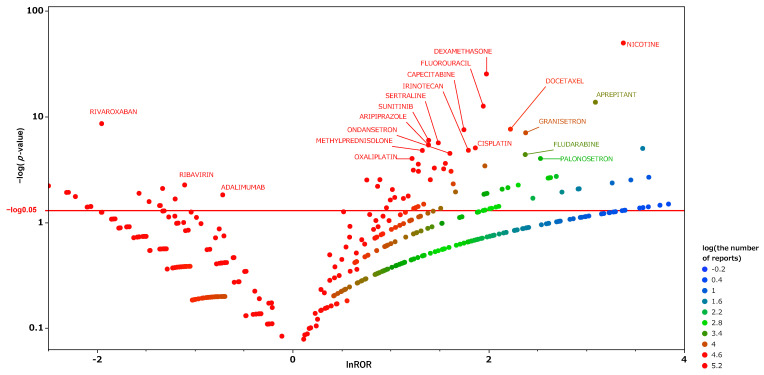
The same as Figure 3, except that this is a scatter plot for the male table.

**Figure 5:**
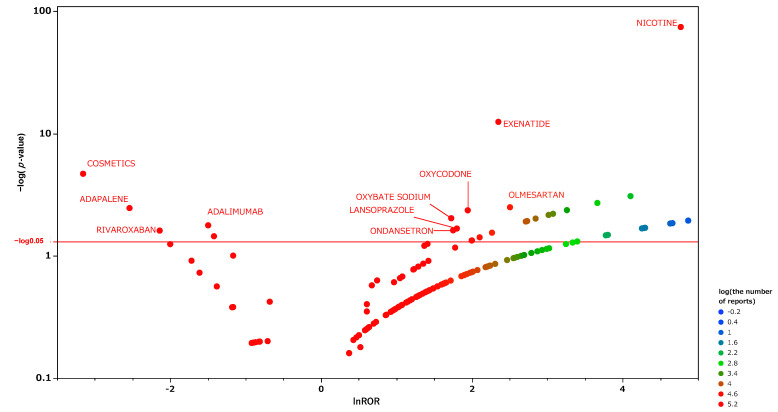
The same as Figure 3, except that this is a scatter plot for the female table.

We produced an encyclopedic scatter plot of the relationship between the reporting odds ratio (ROR) and the significant differences in order to examine medicines suspected of causing hiccups and other drugs. This scatter plot, produced from the analysis tables, is shown in Figure 3, and the corresponding scatter plots obtained using the separate tables for males and females are shown in Figure 4 and Figure 5, respectively. In each figure, the *x*-axis represents the natural logarithm of the reporting odds ratio (lnROR). The positive x-direction indicates that adverse effects associated with hiccups were reported more than other adverse effects. The *y*-axis represents the negative logarithm of the *p*-value [−log_10_(*p*-value)] from Fisher’s exact test. The positive y-direction represents a strongly significant difference. In other words, for medications plotted in the upper right side of this figure, both the ROR and the significant differences were large. We defined such medications as high-signal drugs. The data for each scatter plot are attached as supplementary data (Supplementary S1, total; Supplementary S2, male; Supplementary S3, female).

From these data, we extracted drugs with reporting odds ratios greater than unity, Fisher’s exact test *p*-values of less than 0.001, and more than 5000 reported adverse effects. We then performed multivariate analyses on those drugs with the patient information. The results are shown in Table 2. The results identified male gender, older age, and lower body weight as risk factors for hiccups in the patient information. Fifteen drugs were extracted as risk factors, including nicotine, fluorouracil, sunitinib, aripiprazole, exenatide, antithymocyte immunoglobulin, sertraline, capecitabine, gemcitabine, granisetron, dexamethasone, bortezomib, sorafenib, cisplatin, and methylprednisolone. The ROC-AUC, which indicates the prediction accuracy of the model value, was 0.82.

### 2.4. Risk Factors for Hiccups in Males

From the scatter plot of the male data table (Figure 4), nicotine, dexamethasone, and fluorouracil appear as high-signal drugs. Exenatide, an antidiabetic drug observed as a high-signal drug in all data tables, was not observed as such in men.

We extracted drugs with an ROR greater than unity and a Fisher’s exact test *p*-value of less than 0.001, with more than 3000 reported adverse effects. We performed multivariate analyses on those drugs with gender and weight in the male data table. The results are shown in Table 3. The results identified older age and lower body weight as risk factors for hiccups in the male patient information. Twelve drugs, including anticancer drugs, supportive-care drugs, antipsychotics, and steroids, were extracted as independent variables. These 12 drugs included nicotine, aripiprazole, fluorouracil, sertraline, antithymocyte immunoglobulin, sunitinib, capecitabine, gemcitabine, dexamethasone, methylprednisolone, bortezomib, and cisplatin. The ROC-AUC, which indicates the prediction accuracy of the model value, was 0.72.

### 2.5. Risk Factors for Hiccups in Females

From the scatter plot for the female group, nicotine showed a particularly high signal. Unlike the male group, anticancer drugs, steroids, and psychotropic drugs were not extracted as high signal drugs in the female group. We extracted drugs with an ROR greater than unity and a Fisher’s exact test *p*-value of <0.001, with more than 5000 reported adverse effects. We performed multivariate analyses on those drugs using gender and weight from the female data table. The results are shown in Table 4. From the patient information, only age was extracted as a risk factor for hiccups. Four drugs—nicotine, dasabuvir, exenatide, and sodium oxybate—were extracted as independent risk factors for females.

## 3. Discussion

FAERS is a global database of adverse-event reports from the U.S., Japan, EU countries, etc. This database is much larger than other adverse drug reaction databases. In the analysis table used in this study, the United States had the highest number of reports, 756,468 (55.1%), followed by France with 99,088 (7.2%), and Japan with 96,960 (7.1%).

The adverse-event reporting systems are different in each country. In JADER, Japan’s Adverse Drug Event Report database, medical practitioners and pharmaceutical companies report adverse drug reactions. On the other hand, in FAERS, which we used in this study, patients can report their own side effects. It is thus necessary to keep in mind not only the size of the database but also the characteristics of the data. Adverse drug reactions reported only by medical personnel may be more severe cases. On the other hand, minor side effects may be included if they are reported by the patients themselves.

In this study, the number of hiccups reports was 1454 (843 cases), and the hiccup reporting rate was about 0.06% of all adverse drug event reports. Both the number of cases and the percentage of reports of hiccups were higher for FAERS. There is no point in making simple comparisons between the JADER and FAERS data because they were obtained at different times. The reason for the larger number of cases in FAERS is because it is a larger data set than JADER and because there are differences in the systems for reporting adverse drug events. As hiccups are not a life-threatening symptom, they are rarely reported by medical professionals, but they tend to be more severe when they are so reported. On the other hand, because these are unpleasant symptoms for the patients, the number of self-reports is higher, and minor hiccups may be included in reports.

We also surveyed the countries reporting on hiccups and found some reporting bias but no findings of regional differences. In any case, it is difficult to examine regional differences in the incidence of hiccups from the FAERS data because of the large difference in the number of reports in each country.

From the results of this study, approximately 74% of the reports of hiccups involved men. The incidence of hiccups in males tends to be less than the results obtained using JADER [11]. As mentioned earlier, because FAERS includes reports from non-medical patients, non-serious side effects may also be reported. This suggests that severe hiccups (as reported by medical staff) are male-dominated, whereas non-serious hiccups show less gender difference. The results of the multivariate analysis showed that, as in previous reports, male gender was extracted as an independent risk factor for hiccups. A study comparing hiccups of central nervous system (CNS) and non-CNS origin reported that male predominance persisted in hiccups of non-CNS origin. Drug-induced hiccups are peripheral hiccups, and the threshold of nerve excitation associated with hiccups may be lower in males [6]. This study revealed that hiccups as a side effect are more likely to occur in males, and it is suspected that the effects of the drugs differ by gender. There are gender differences in effects and adverse effects, and drug-induced hiccups may also be influenced by gender differences in drug dosage, absorption, metabolism, and excretion. Further research to elucidate the mechanism of drug-induced hiccups may lead to new insights into the gender differences of the effects of drugs. Hiccups may also develop predominantly in males as they get older [6]. In this study, we examined age and hiccup incidence and found that male incidence did not change considerably with age (Figure 2); thus, we did not find an association between age and gender in drug-induced hiccups. No report has yet elucidated the mechanism of the relationship between hiccups and gender differences. In addition, some studies have reported that the neurotransmitters GABA and serotonin are involved in hiccups [4,15]. There has also been a report of gender differences in the receptors and effects of GABA and serotonin [16]. Neurotransmitters and other neural mechanisms may also be involved in gender differences in hiccups. The effects of each suspect drug on receptor activity and neurotransmitter pathways were not elucidated in this study, and future studies in this regard are expected.

In the results of multivariate analysis, older age was extracted as a risk factor for hiccups. A retrospective study of hiccups in hospitalized patients reported a trend towards a higher incidence of hiccups in infants with low birth weights and in patients after the age of 40 years [12]. In the present study, there was only one report of hiccups in infants, and infancy was not considered to be a risk factor in drug-induced hiccups. In older patients, the increase in medications containing drugs that induce hiccups may be a risk factor for hiccups. Some studies have reported that hiccups are associated with GABA receptors [17], and GABA receptors can reportedly change their action with age [18]. Thus, age-related changes in receptors may be the reason why older age emerged as a risk factor for hiccups.

In this study, we found no association between hiccups and body weight, which is among the physical information included in FAERS. In previous studies, we have reported a relationship between low BMI and tall stature and hiccups [11,12]. The FAERS used in this study was reported from several countries, and generalizing about the association between hiccups and physical information, such as weight, is challenging. If a substantial amount of data can be collated regarding hiccups in healthy people, we may be able to gain new knowledge about gender differences and physical information.

We performed an exhaustive univariate analysis of the association between hiccups and drugs using FAERS. We visualized the results using scatter plots (Figure 3). As hiccups are known to have gender differences, we further analyzed them separately for males and females, and visualized these data as well (Figure 4 and Figure 5). The results show that there is a difference between men and women in the medications suspected of inducing hiccups. From the scatter plots, nicotine was detected for both men and women as a suspect drug for hiccups with a high signal. Anticancer drugs, steroids, and antipsychotics were extracted with high signals in the male group, while antiviral drugs and antidiabetic drugs were extracted with high signals in the female group. In multivariate analyses, we were able to extract independent risk factors for hiccups in the separate groups of men and women. This is the first report to examine the suspected drugs and patient’s information by gender.

As noted above, nicotine has been revealed as an important suspect drug for hiccups in both men and women, although there is only one case report of an association between nicotine and hiccups [19]. The present report is the first to identify it as a risk factor for hiccups. The JADER database showed no association between nicotine and hiccups. This is because nicotine is sold as a stop-smoking medication as an over-the-counter drug and is not included in the Japanese database.

It is empirically known that smoking can cause hiccups, but the mechanism responsible for this is not clear. The inhalation of substances from smoking may irritate the lungs and induce hiccups as a vagal reflex. However, the sources of nicotine in FAERS are pharmaceutical gums or patches. Thus, the present study shows that the intake of nicotine other than by smoking can induce hiccups. This suggests that nicotine itself or its metabolites may be causing hiccups in the body.

Although there have been no reports on the mechanism by which nicotine induces hiccups, it is possible to consider some hypotheses. Nicotine facilitates autonomic ganglion and neuromuscular-junction transmission by nicotinic acetylcholine (ACh) receptors (nAChRs). In addition, it causes membrane depolarization of many neurons in the central nervous system (CNS) and acts on presynaptic terminals to promote the release of transmitters [20]. Furthermore, nicotine is known to act on nicotinic receptors on dopamine nerve endings to stimulate dopamine release [21]. The hiccup is known as a reflex arc, and its pathways have been identified. The afferent pathway is the pharyngeal branch of the glossopharyngeal nerve, and hiccups are centered in the reticular formation near the nucleus ambiguous. In contrast, the efferent pathway is represented by the diaphragmatic nerve and the vagus nerve [3]. Baclofen, a GABA_B_ agonist, has been shown to suppress hiccups, suggesting that the hiccup center is inhibited by GABA [4]. These findings suggest that nicotine induces hiccups by releasing dopamine, which is involved in GABAergic inhibition. In addition, hiccups are suppressed by chlorpromazine and metoclopramide, which are dopamine D_2_ blockers, suggesting that a central dopamine receptor agonist is involved in hiccups [22]. This suggests that nicotine may induce hiccups by acting on nicotinic acetylcholine receptors in the brain, releasing dopamine, and acting directly on dopamine receptors.

In this study, there was no gender difference in the hiccups induced by nicotine. Although most drug-induced hiccups are known to be male-dominated, there was no gender difference in nicotine-induced hiccups. The mechanism by which nicotine induces hiccups may thus be different from that of other drugs. Moreover, due to the characteristics of FAERS, which includes minor symptoms, it is possible that severe hiccups may be male-dominated, while nicotine-induced hiccups are minor symptoms and therefore do not show a gender difference. Further research is needed on the relationship between nicotine and hiccups, including the above hypothesis.

The scatter plot of the results from the univariate analysis showed that drugs used in chemotherapy, antipsychotics, and nicotine had high signals (Figure 4). In a multivariate analysis that included patient information, the independent risk factors were identified as nicotine, anticancer drugs, psychotropic drugs, steroids, and immunoglobulin products.

There have been some reports on the relationship between psychotropic drugs and hiccups. Chlorpromazine is the only treatment drug for hiccups approved by the FDA, and it has been reported to be effective in treating hiccups [23]. Chlorpromazine is a dopamine receptor blocker and has been used for many years as a treatment for schizophrenia. As chlorpromazine is known to have a high affinity for dopamine D_2_ and D_3_ receptors [24], the onset of hiccups is believed to be related to dopamine receptors. There has also been a report of hiccups induced by levodopa, a dopamine preparation [25]. However, the mechanism of the relationship between dopamine receptors and hiccups has not been clarified. There have also been reports of an association between hiccups and serotonin receptors [15]. These findings suggest that the pathogenesis of hiccups involves multisystemic neurotransmission. In this study, aripiprazole, a partial agonist of dopamine receptors, and sertraline, a selective serotonin reuptake inhibitor (SSRI), were risk factors for hiccups. In addition, there have been some reports of aripiprazole inducing hiccups [7,26,27,28]. Aripiprazole is an antagonist for 5-HT_1A_ receptors, which may be involved in inducing hiccups. Alefishat et al. compared the affinity of the receptors for aripiprazole and for the structurally similar brexpiprazole—which has not been reported to induce hiccups—but the results were not conclusive for identifying the receptors associated with hiccups [29]. There are a few reports of sertraline inducing hiccups [30,31]. Sertraline selectively binds to the serotonin transporter with a low affinity for other transporters and receptors [32]. This result suggests that sertraline may be associated with the induction of hiccups, but the mechanism is not clear. Psychotropic drugs have been widely reported both as treatments and as inducing agents for hiccups. It is also interesting to note that in the present study, these were only risk factors for hiccups in males. In the future, it will be necessary to accumulate cases and conduct further molecular-structure studies of these drugs.

In this study, the steroids methylprednisolone and dexamethasone were identified as risk factors for hiccups in men. Many reports exist on the relationship between steroids and hiccups, yet the mechanism is not clear [9,11,33]. In a study using JADER, a Japanese database, dexamethasone was extracted among steroids as a strong inducer for hiccups [11].

Dexamethasone and methylprednisolone are effective in chemotherapy-induced nausea and vomiting (CINV), and they are frequently used as an adjunctive therapy with drugs having strong emetic actions. Chu et al. [34] have speculated that the following effects of steroids may be relevant to the mechanism of CINV suppression: (1) an anti-inflammatory effect; (2) direct central action at the solitary tract nucleus; (3) interaction with the neurotransmitter serotonin and the receptor proteins tachykinin NK1 and NK2, alpha-adrenaline, etc.; (4) maintaining the normal physiological functions of organs and systems; (5) regulation of the hypothalamic–pituitary–adrenal axis; and (6) reducing pain and the concomitant use of opioids, which in turn reduces opioid-related nausea and vomiting. It has also been reported that the center of hiccups is located in the nucleus of the solitary tract in the medulla oblongata [3], therefore, the direct effect of steroids on the medulla oblongata may induce hiccups. It is also known that serotonin is involved in the induction and suppression of hiccups [15], and the effect of steroids on serotonin receptors may be involved in the induction of hiccups.

As a method to deal with chemotherapy-induced hiccups, Lee et al. reported that steroid rotation performed by changing dexamethasone to methylprednisolone can reduce hiccups [10]. In that report, a small number of patients still experienced hiccups after the change to methylprednisolone, which is consistent with the present finding that both dexamethasone and methylprednisolone are risk factors for hiccups. The results of the analysis of the male data table also supports the study by Lee et al. in that the number of reports of hiccups for dexamethasone was 51 out of 20,095 reports (0.25%) compared with 16 out of 12,024 (0.13%) for methylprednisolone. Differences in the permeability of the blood–brain barrier (BBB) have been considered as a reason for the high risk of hiccups associated with dexamethasone. It has been argued that steroids entering the CNS may lower the threshold for nerve stimulation in the hiccup center [10]. In addition, Kondo et al. have discussed the central effects of dexamethasone, suggesting that the GABAergic and antagonistic effects are responsible for inducing hiccups [35].

In recent years, the involvement of nuclear receptors as a mechanism for the action of steroids has been reported, and research is progressing. After passing through the cell membrane, a steroid binds to the corticoid receptors in the cytoplasm. Steroid-bound corticoid receptors are thought to migrate into the nucleus and regulate the expression of target genes at the level of transcription factors. Glucocorticoids are highly fat-soluble, and they pass through the BBB and bind to corticosteroid receptors in the brain. There are two types of corticoid receptors, glucocorticoid (GR) and mineralocorticoid (MR), and it is known that their effects differ depending on the binding affinity of the corticoid [36]. Although there have been no reports on the relationship between hiccups and corticoid receptors, steroids may act on corticoid receptors in the brain and induce hiccups. It is also possible that differences in the potency and affinity of steroids may cause differences in the induction of hiccups.

This study suggests the new finding that the steroids dexamethasone and methylprednisolone are male-specific risk factors. In the future, it will be necessary to study the effects of drugs on BBB permeability, GABA receptors, serotonin receptors, and nuclear receptors in the brain from various aspects such as gender, the chemical structure of drugs, and affinity. By clarifying these factors, we hope to help elucidate the mechanism by which steroids induce hiccups.

There have been many reports on anticancer drugs and hiccups. In this study, the anticancer drugs extracted as risk factors for hiccups in men were fluorouracil, sunitinib, capecitabine, gemcitabine, bortezomib, and cisplatin.

Anticancer drugs that have been reported to induce hiccups in the past include fluorouracil [11,35], gemcitabine [13], and cisplatin [8,37]. A mechanism that may be involved in the induction of hiccups by cisplatin is the stimulation of the vagus nerve due to the release of serotonin because of the stimulation of the enterochromaffin cells [38]. Fluorouracil, capecitabine, and gemcitabine are the antimetabolite and pyrimidine-analog families of medications. There have been limited numbers of reports on hiccups and these drugs, and no reports support a causal relationship. These are drugs that cause nausea, vomiting, and diarrhea as adverse events. These symptoms may stimulate the afferent tracts of the vagus nerve and induce hiccups. In addition, fluorouracil, capecitabine, and gemcitabine are highly emetogenic drugs, and they are often used in combination with steroids, such as dexamethasone, for CINV prevention; thus, the association with dexamethasone-induced hiccups should also be considered.

There are no published reports on the relationship between sunitinib and hiccups, and the risk factors and mechanisms are unknown. Sunitinib side effects have been reported to be related to gastrointestinal disorders such as nausea and vomiting, and there is a possibility of hiccups related to CINV. We also need to consider the mechanism by which molecularly targeted drugs induce hiccups. In the future, it will be necessary to accumulate cases.

Furthermore, the present study extracted bortezomib as a risk factor for hiccups in men. Bortezomib is a proteasome inhibitor used to treat multiple myeloma and mantle-cell lymphoma. There have been no reports of hiccups with bortezomib. Bortezomib is sometimes used with dexamethasone in chemotherapy, which may be associated with hiccup induction. The results of this analysis showed no internal correlation between dexamethasone and bortezomib. It is possible that the mechanism of inducing hiccups is related to other adverse events or that there is some novel mechanism.

This is the first report of immunoglobulin as a risk factor for male hiccups. According to drug information for ATGAM, an immunoglobulin, hiccups have been reported in 0.4% of patients [39]. The mechanism is not known. Nausea (4.2%) and vomiting (3.4%) have been reported as side effects of immunoglobulin preparations [39]. Immunoglobulin-induced hiccups also may be related to nausea and vomiting [13]. There is also the possibility of a new mechanism or a primary disease that is treated with immunoglobulin.

The risk factors for inducing hiccups in women were nicotine, dasabuvir, exenatide, sodium oxybate, and age. Nicotine was mentioned above as a common risk factor for both men and women.

Dasabuvir is one of the antiviral drugs used in the treatment of hepatitis C virus (HCV) infection. Dasabuvir acts as an NS5B polymerase inhibitor, and it is used in combination with the fixed-dose combination of ombitasvir, paritaprevir, and ritonavir [40]. There are no reports on dasabuvir and hiccups, and the details are unknown. Nausea and diarrhea have been reported as side effects of combination therapy for HCV [40], and these symptoms may have triggered hiccups. It is also possible that there is some relationship between NS5B polymerase inhibitor and hiccups, but there is no clear information at this time.

Exenatide is a GLP-1 receptor agonist used in the treatment of diabetes mellitus. Exenatide is effective in promoting blood glucose-dependent insulin secretion from the islet of Langerhans beta cells in the pancreas, inhibiting glucagon secretion from the same alpha cells, and decreasing the rate of gastric emptying. In addition, this GLP-1 receptor agonist is used as an anti-obesity drug in the U.S; this may be related to the gender differences in the number of side effects reported. There have been no reports of exenatide inducing hiccups. GLP-1 is classified as either short-acting or long-acting, depending on the duration of action, and it has been pointed out that the mechanism of action for improving blood glucose is different for the two classes [41]. Exenatide is classified as a short-acting drug, and it is known to have gastrointestinal side effects associated with delayed gastric emptying, which may induce hiccups. It may also induce metabolic hiccups due to the underlying diabetes mellitus. This is the first time that GLP-1 has been reported as a risk factor for inducing hiccups in women.

Sodium oxybate is a sodium salt of γ-hydroxybutyric acid (GHB), and is used as a treatment for narcolepsy. Gamma-hydroxybutyrate (GHB) is a short-chain fatty acid endogenously produced within the CNS, and it acts as a precursor to and metabolite of the inhibitory neurotransmitter GABA. GHB is related to these effects due to the increase in dopamine by GABA_B_ receptors in the mesocorticolimbic circuit [42]. Although it has not been reported to directly induce hiccups, it may induce them due to the activation of dopamine-related nerves. The induction of hiccups by sodium oxybate is a new report. Further studies are needed to determine why it was extracted as a side effect in women and its relationship to GABA and dopamine.

This study used data from a database, therefore some limitations should be considered. As FAERS is based on a self-reporting system, some biases should be considered. Spontaneous reports of adverse drug events are particularly useful in detecting rare and serious adverse drug reactions, and they are an important source of information in assessing the safety of a drug. Mild adverse effects are only occasionally reported, with severe cases being reported more often. This is a known reporting bias, which is characteristic of a self-reported database [43]. FAERS is thus likely to have a stronger reporting bias because the patients who take the drugs are also the reporters. If the patient is taking several drugs, it is possible that a reaction may actually be caused by an entirely different drug and not the one that is designated as the culprit. The failure to consider that other drugs may be responsible for the reaction may have been a potential source of bias when calculating the RORs [44]. Selection bias and confounding also need to be taken into consideration. The adverse events database is for people who use medications; thus, it does not include healthy people who do not use medications. It is also possible that as people age, the number of drugs they take increases, and this increases the relative proportion of people who develop side effects. In addition, although we did not show the results of the statistical tests for the differences between males and females, we were able to observe significant differences based on the comparison of the odds ratios.

In this study, we examined the number of reports, along with the ROR obtained from univariate analysis and the *p*-value results obtained from the exact test, in detecting signals. We treated the RORs semi-quantitatively, avoiding simple comparisons. This is based on the hypothesis that signal-detection indicators that are supported as highly significant by the number of reports and *p*-values have excellent credibility. Authors should discuss their results and how they can be interpreted from the perspectives of previous studies and working hypotheses. Their findings and implications should be discussed in the broadest possible context, and future research directions might also be highlighted.

## 4. Materials and Methods

### 4.1. Data Source

In this study, we used FAERS, a worldwide database published by the U.S. FDA of adverse events involving medicines that have been collected from all over the world, including Japan. FAERS is a large-scale database that comprehensively links drugs, their side effects, and patient information. We first downloaded the FAERS database from the FDA website [14], and we used our data-cleaning method to generate the data source. The extraction period covered 11,810,863 adverse drug reactions reported between the first quarter of 2004 and the first quarter of 2020.

### 4.2. Definitions of the Terms Adverse-Event and Suspect Medicine

The adverse-event terms appearing in the FAERS database are based on the Preferred Terms found in the Medical Dictionary for Regulatory Activities (MedDRA) [45]. In this study, we extracted the Preferred Term “Hiccups” from the MedDRA version 18.1. In each case, we classified the contributions of the adverse events involving administered medications into four categories: “primary suspect drug”, “secondary suspect drug”, “concomitant”, and “interaction”. We extracted all cases that were classified as such as suspect medicine.

### 4.3. Production of the Data Analysis Table

**Figure 6:**
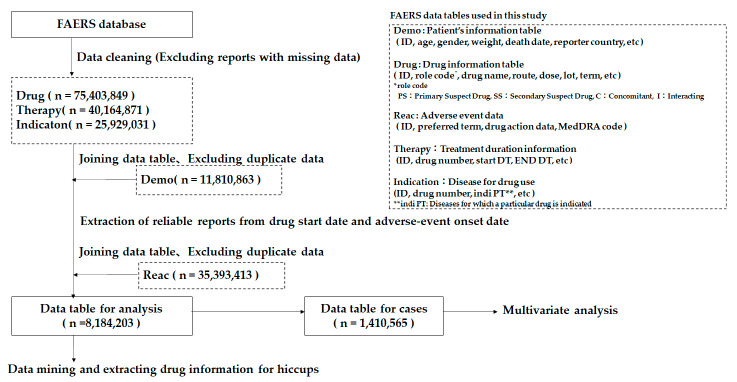
Production of a data analysis table.

FAERS consists of seven tables: (1) DEMO, (2) DRUG, (3) INDICATION, (4) OUTCOME, (5) REACTION, (6) REPORT SOURCES, and (7) THERAPY. Figure 6 shows the items included in each table and the number of reports obtained between the first quarter of 2004 and the first quarter of 2020. We used the five tables (1)–(3), (5) and (7) above for the analyses reported in this study.

We removed duplicated data from the DRUG, INDICATION, REACTION, and THERAPY tables. We then connected the DEMO table to the DRUG, INDICATION, and THERAPY tables using the primary ID number for each adverse effect case. Figure 6 shows the flowchart we used to construct the data analysis tables. To clarify the relationship between drug use and side effects, we checked the date of drug initiation, the date of drug termination, and the date of side effect occurrence. From these data, we only extracted data in which side effects occurred between the start and end dates of the drug administration [46]. Finally, we connected a REACTION table using the primary ID. We defined this table as the data table for the analysis.

### 4.4. Extraction of Suspect Drugs for Hiccups Using Data Mining Methods

**Figure 7:**
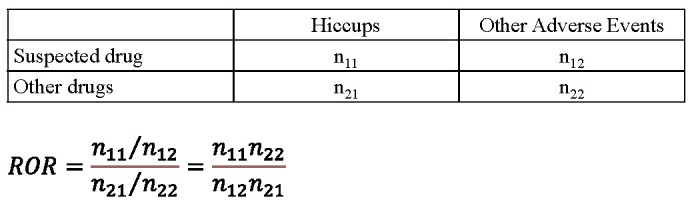
Cross-tabulation and formula for the ROR of hiccups. The cross-tabulation is structured using reports of suspected drugs, all other reports, reports with hiccups, and reports without hiccups (n_11–22_ indicates the number of cases). The reporting odds ratio (ROR) was calculated as shown.

We compiled a cross-tabulation based on two classifications: the presence or absence of hiccups and the presence or absence of the suspected drug (Figure 7). We also calculated the *p*-value through Fisher’s exact test and the ROR. The ROR is the ratio of one report of a specific adverse effect versus all other adverse effects for a given drug to the reported odds for all other drugs present in the database. We considered that a signal exists when the lower limit of the 95% confidence interval of the ROR is greater than unity.

We then compiled a scatterplot (volcano plot) by plotting the negative logarithm of the *p*-value (−Log_10_*p*) from Fisher’s exact test on the *y*-axis and the natural logarithm of the ROR (lnROR) on the *x*-axis.

### 4.5. Relationship between Patient Information and Hiccups

We conducted a univariate analysis of the patient information (gender, age, and weight) included in FAERS, with the presence or absence of hiccups as the objective variable. We treated gender as a nominal variable and performed Fisher’s exact test. We also analyzed age and weight, conducted a *t*-test, and calculated *p*-values. We treated these data as absolute numbers and analyzed them as continuous variables. Many items had missing values, and we analyzed only those that did not include missing values. We treated weights heavier than 400 kg and ages greater than 119 years old as outliers and excluded them from our analysis.

### 4.6. Multivariate Analysis

To extract risk factors for hiccups, we performed a nominal logistic regression analysis, with the presence or absence of hiccups as the response variable. The explanatory variables were the important suspect drugs obtained in the univariate analysis and the patient information included in FAERS. To treat gender as one of the objective variables, it was incorporated into the variables as a nominal variable (male: 1, female: 0). Important suspect drugs were those with more than 3000 adverse drug-reaction reports, Fisher’s exact test *p*-values of 0.001 or less, and reporting odds ratios greater than unity.

### 4.7. Subgroup Analysis of the Two Separate Groups of Men and Women

We divided the analysis data table created in Section 2.3 into male and female data tables. In this analysis, we excluded data described by a gender other than male or female. In each group, we extracted risk factors using the method described in Section 2.4 and Section 2.5. The important suspected drugs in the male table were those with more than 3000 adverse drug-reaction reports, Fisher’s exact test *p*-values of 0.001 or less, and odds ratios greater than unity. The most important suspected drugs in the female group were those with more than 5000 adverse drug-reaction reports, Fisher’s exact test *p*-values of less than 0.05, and odds ratios greater than unity.

### 4.8. Statistical Analysis

We calculated means (±standard deviations) for all the continuous variables. We considered a *p*-value < 0.05 to be significant. We estimated the internal correlation using the pairwise method. When the square of Spearman’s rank-order correlation coefficient [ρ2] was greater than 0.9, we concluded that there was an internal correlation. When there was no internal correlation, we treated these items as independent factors. All analyses were performed with JMP1Pro14 (SAS Institute Inc., Cary, NC, USA).

## 5. Conclusions

In this study, we obtained new findings on risk factors for hiccups using FAERS, a worldwide database. There are many unanswered questions about drug-induced hiccups, and this study may help to elucidate the mechanism of drug-induced hiccups because it looked at the suspect drugs comprehensively. In addition, this is the first study to show that suspected drugs differ between male and female groups. This is a very important finding for the pathogenesis of drug-induced hiccups, and will aid future research on hiccups.

In this study, we were able to report new suspect drugs for hiccups. In particular, nicotine was extracted as a common suspect drug for both males and females, which may indicate a new pathway for hiccups. We believe that further molecular–chemical investigations of the suspect drugs will lead to the elucidation of the mechanism responsible for drug-induced hiccups.

We were able to show that the data mining method used in this study is a useful method for extracting suspect drugs for side effects that are rarely reported and for understanding patient characteristics.

Elucidation of the risk factors and of the mechanisms that cause the onset and suppression of hiccups can contribute to a patient’s QOL in terms of prevention and treatment.

## Figures and Tables

**Table 1:** Patient information.

a. All data table				
	Number of cases	Hiccups (N = 843)	Non-hiccups (N = 1,409,722)	*p*-Value
Gender (M/F)	1,348,038	622/193	544,997/803,041	<0.001
age_YR	1,202,581	59.28 ± 0.76	54.63 ± 0.04	<0.001
wt_KG	541,174	74.37 ± 1.36	73.12 ± 0.01	0.3561
b. Male data table				
	Number of cases	Hiccups (N = 622)	Non-hiccups (N = 544,997)	*p*-Value
age_YR	483,580	59.35 ± 0.86	57.59 ± 0.03	0.0391
wt_KG	231,008	75.81 ± 1.58	78.51 ± 0.06	0.0882
c. Female data table				
	Number of cases	Hiccups (N = 193)	Non-hiccups (N = 803,041)	*p*-Value
age_YR	709,459	59.02 ± 1.59	52.61 ± 0.02	<0.001
wt_KG	307,508	69.72 ± 2.88	69.08 ± 0.05	0.8229

Each item included missing values; however, the analyses were performed using data without missing values. The sum of the male and female data tables stratified by gender does not match the sum of all the data tables.

**Table 2:** Results of a multiple logistic regression.

	Odds Ratio	CI (95%)
NICOTINE	93.38 *	69.92–124.72
FLUOROURACIL	8.22 *	4.38–15.43
SUNITINIB	8.19 *	4.67–14.38
ARIPIPRAZOLE	8.14 *	3.99–16.60
EXENATIDE	7.52 *	3.51–16.11
ANTITHYMOCYTE IMMUNOGLOBULIN	6.89 *	2.75–17.24
SERTRALINE	6.32 *	3.11–12.84
CAPECITABINE	6.01 *	3.36–10.76
Gender (male: 1, female: 0)	5.19 *	4.06–6.63
GEMCITABINE	4.34 *	1.98–9.48
GRANISETRON	4.01 *	1.89–8.51
DEXAMETHASONE	3.48 *	2.09–5.79
BORTEZOMIB	3.47 *	1.62–7.46
SORAFENIB	3.20 *	1.50–6.81
CISPLATIN	2.62 *	1.17–5.87
METHYLPREDNISOLONE	2.30 *	1.06–5.01
PREDNISONE	2.02	0.75–5.48
BEVACIZUMAB	1.76	0.85–3.64
IRINOTECAN	1.70	0.74–3.87
ONDANSETRON	1.69	0.72–4.00
age_YR	1.01 *	1.003–1.014
wt_KG	0.99 *	0.989–0.998
OXALIPLATIN	0.91	0.43–1.93
LENALIDOMIDE	0.51	0.18–1.42
FOLINIC ACID	0.38	0.11–1.31

*: indicates a significant odds ratio.

**Table 3:** Results of a multiple logistic regression using the male data table.

	Odds Ratio	CI (95%)
NICOTINE	61.73 *	42.16–90.38
ARIPIPRAZOLE	9.26 *	4.52–19.01
FLUOROURACIL	8.90 *	4.65–17.05
SERTRALINE	8.65 *	4.24–17.64
ANTITHYMOCYTE IMMUNOGLOBULIN	8.57 *	3.54–20.77
SUNITINIB	8.33 *	4.63–14.99
CAPECITABINE	6.97 *	3.79–12.80
GEMCITABINE	5.12 *	2.32–11.30
DEXAMETHASONE	4.47 *	2.83–7.06
METHYLPREDNISOLONE	2.79 *	1.30–5.98
BORTEZOMIB	2.59 *	1.15–5.83
CISPLATIN	2.51 *	1.09–5.75
PREDNISONE	2.31	0.86–6.25
age_YR	2.23 *	1.09–4.56
DOCETAXEL	1.88	0.44–8.09
IRINOTECAN	1.86	0.80–4.31
BEVACIZUMAB	1.71	0.79–3.69
ONDANSETRON	1.67	0.70–4.00
OXALIPLATIN	0.97	0.45–2.06
FOLINIC ACID	0.43	0.12–1.48
wt_KG	0.10 *	0.02–0.59

*: indicates a significant odds ratio.

**Table 4:** Results of a multiple logistic regression analysis using the female data table.

	Odds Ratio	CI (95%)
NICOTINE	192.16 *	120.75–305.79
DASABUVIR	23.65 *	5.73–97.67
EXENATIDE	11.38 *	3.46–37.44
SODIUM OXYBATE	11.17 *	1.53–81.85
age_YR	1.01 *	1.001–1.026
wt_KG	1.00	0.988–1.009

*: indicates a significant odds ratio.

## Data Availability

Data are contained within the article.

## Supplementary Materials

The following supporting information can be downloaded at: https://www.mdpi.com/article/10.3390/ph15010027/s1, Supplementary S1: Scatter plot data in the total table; Supplementary S2: Scatter plot data in the male table; Supplementary S3: Scatter plot data in the female table.
